# Doxycycline-induced photosensitivity in patients treated for erythema migrans

**DOI:** 10.1186/s12879-018-3270-y

**Published:** 2018-08-03

**Authors:** Maša Velušček, Fajko F. Bajrović, Franc Strle, Daša Stupica

**Affiliations:** 1 Department of Infectious Diseases, University Medical Centre Ljubljana, Japljeva 2, 1525 Ljubljana, Slovenia; 2 Department of Neurology, University Medical Centre Ljubljana, Zaloška 2, 1000 Ljubljana, Slovenia; 3 Faculty of Medicine, University of Ljubljana, Vrazov trg 2, 1104 Ljubljana, Slovenia

**Keywords:** Doxycycline, Photosensitivity, Erythema migrans, Tick-borne disease

## Abstract

**Background:**

Doxycycline is one of the recommended antibiotics for treating erythema migrans (EM). Since EM predominantly occurs during summer, the potential of doxycycline to induce photosensitivity is of concern. In studies on the efficacy of doxycycline for treating relatively small numbers of patients with EM, the reported frequency of photosensitivity has varied from none to 15%. The aim of this study was to elucidate the frequency and clinical symptoms of doxycycline-induced photosensitivity in a large cohort of patients with EM treated in a single medical centre.

**Methods:**

Prospectively collected data on adverse events were analysed in adult patients with EM treated with doxycycline 100 mg twice daily for 10–15 days.

**Results:**

Photosensitivity reactions ranging from itching and burning sensations to transient mild erythema of sun-exposed skin were documented in 16/858 (1.9, 95% CI 1.1–3.0%) patients and appeared from June to October with highest frequency in July. These adverse events were more frequent in patients treated for 14 or 15 days (16/750 [2.1%]; 95% CI 1.2–3.4%) than in those treated for 10 days (0/108 [0%]; 95% CI 0.0–3.4%); however, the difference was not significant (*P* = 0.24). Women were more often affected than men (13/475 [2.7%], 95% CI 1.5–4.6% versus 3/383 [0.8%], 95% CI 0.2–2.3%; *P* = 0.04). Of the 16 patients who developed photosensitivity, 13 did not adhere to the recommendation to avoid sun exposure. None of the patients had any long-term sequelae of photosensitivity.

**Conclusions:**

Photosensitivity reactions in adult patients with EM treated with doxycycline 100 mg twice daily for 10–15 days occurred rarely, were not severe, and had no long-term sequelae.

**Trial registration:**

Registered at http://clinicaltrials.gov, Identifiers NCT00910715, May 28th 2009, NCT01163994, July 13th 2010 and NCT03584919, June 19th 2018 retrospectively registered.

## Background

Erythema migrans (EM) is the most frequent manifestation of Lyme borreliosis [1]. In addition to amoxicillin and cefuroxime axetil, oral doxycycline is a recommended treatment regimen for adult patients with EM [2]. Doxycycline has the advantage of its efficacy against *Anaplasma phagocytophilum*, the possible coinfecting bacterium in patients with early Lyme borreliosis [2], but its major drawback is potential photosensitization [3, 4], which is of particular concern because EM predominantly occurs during summer. Patients with EM who are prescribed doxycycline are therefore advised to avoid exposure to the sun while receiving therapy [2].

In studies on the treatment efficacy of doxycycline in patients with EM, reported photosensitivity ranged from none (0/108) to 15% (9/60) (Table 1) [3–14]. The reasons for this wide range are not clear and cannot be explained solely by variations in treatment duration or dosing of doxycycline in different treatment trials (Table 1). Variations in the advice to patients on sun restriction and in monitoring adverse events at different centres may be implicated. Further, the relatively small numbers of patients evaluated in individual trials probably also contributes to such pronounced disparities. In these studies, clinical symptoms of photosensitivity were not described. According to a recent review they can vary from a light sunburn-like sensation (burning, erythema) to large-area photodermatitis, and even onycholysis [15].

**Table 1:** Data on photosensitivity in doxycycline-treated patients with erythema migrans in Europe (Eu) and the United States (US)

First author, year	Eu/ US	Number of patients (% of female)	Doxycycline		Patients (%) with photosensitivity
			Dosage	Days	
Dattwyler, 1990 [7]	US	37 (49%)	100 mg twice daily	21	1 (2.7%)
Nadelman, 1992 [4]	US	60 (42%)	100 mg three times daily	20	9 (15%)
Massarotti, 1992 [8]	US	22 (50%)	100 mg twice daily	10–20	1 (4.5%)
Strle, 1992 [13]	Eu	23 (57%)	100 mg twice daily	14	1 (4.3%)
Strle, 1993 [11]	Eu	52 (44%)	100 mg twice daily	14	6 (11.5%)
Luger, 1995 [3]	US	113 (37%)	100 mg three times daily	12–20	7 (6.2%)
Strle, 1996 [12]	Eu	42 (50%)	100 mg twice daily	14	5 (11.9%)
Dattwyler, 1997 [6]	US	72 (39%)	100 mg twice daily	21	9 (12%)
Baršić, 2000 [10]	Eu	40 (52%)	100 mg twice daily	14	0
Wormser, 2003 [5]	US	61 (32%)	100 mg twice daily	10	5 (8.2%)
		59 (40%)		20	2 (3.4%)
Cerar, 2010 [9]	Eu	145 (58%)	100 mg twice daily	15	1 (0.7%)
Stupica, 2012 [14]	Eu	108 (57%)	100 mg twice daily	10	0
		117 (54%)		15	7 (6%)

The aim of the present study was to analyse prospectively collected data on the frequency and intensity of photosensitivity reactions in a large cohort of patients with EM treated with doxycycline in a single centre.

## Methods

Patients ≥18 years, with EM defined according to European criteria [16], treated with doxycycline between June 2006 and October 2015 at the University Medical Centre Ljubljana, Slovenia, and who had been included in three prospective treatment trials were eligible for analysis of doxycycline-induced photosensitivity. Patients received oral doxycycline 100 mg twice daily for 10, 14 or 15 days. In each trial, patients were assigned to treatment groups based on arrival order at the clinic so that every second patient received one of the two treatment options being compared in that particular trial. Patients who were prescribed doxycycline were advised to avoid exposure to the sun. Those who were not compliant with following the prescribed treatment regimen or did not attend the 14-day visit were excluded. At baseline and follow-up at 14 days and at 2, 6, and 12 months, patients were examined physically and were asked an open question about health-related symptoms. On day 14, patients were asked an open question about medication compliance and adverse events.

Numerical data were summarized as medians (interquartile range, IQR) and categorical data as frequencies (%) with two-sided 95% confidence intervals (CI) calculated on exact binomial distributions. Two-tailed Fisher exact tests were used for all bivariate comparisons of proportions and Mann–Whitney U test for comparisons of numeric variables. A *P* value < 0.05 was considered statistically significant.

## Results

A total of 864 patients with EM were evaluated. Among these, three patients were excluded from further analysis after antibiotic treatment was discontinued following an allergic reaction, manifested as maculopapular rash a few days after starting therapy. A further three patients were excluded because they did not attend the 14-day visit. All the remaining 858 patients stated compliance with the prescribed treatment regimen.

Overall, 16/858 (1.9, 95% CI 1.1–3.0%) patients reported photosensitivity reactions: 0/108 (0, 95% CI 0–3.4%), 8/488 (1.6, 95% CI 0.7–3.2%), and 8/262 (3.1, 95% CI 1.3–5.9%) of those who received doxycycline for 10, 14 or 15 days, respectively. Photosensitivity reactions presented from June to October with highest frequency in July (Table 2). The clinical symptoms of photosensitivity developed on the fifth day of therapy in one patient and on the tenth day in another, but exact timing of the onset of photosensitivity reactions for remaining 14 patients was not obtained. Photosensitivity reactions ranged from itching and burning sensations without any change in skin colour in seven patients, to mild erythema of sun-exposed face and extremities in nine patients. Of the 16 patients with photosensitivity reactions, 13 did not adhere to the recommendation to avoid sun exposure completely. One patient was deliberately exposed to the sun while sunbathing, but other 12 patients were exposed only during unavoidable daily activities, such as going to work, which did not exceed one hour per day for an individual patient. None of the patients had any sequelae of photosensitivity at the 2-month visit or later. Women were more often affected than men (13/475 [2.7%], 95% CI 1.5–4.6% versus 3/383 [0.8%], 95% CI 0.2–2.3%; *P* = 0.04). Patients with photosensitivity did not differ from those without photosensitivity according to age (median 43.5 years, IQR 39–55 versus 53 years, IQR 42–62; *P* = 0.08) or according to use of any concomitant medications (6/16 [37.5%] versus 358/842 [42.5%]; *P* = 0.80). None of the patients with photosensitivity was taking medications known to be associated with phototoxicity or to potentially interact with doxycycline, however 24/842 (2.9%) patients without photosensitivity were taking potentially phototoxic medications such as thiazides or phenothiazines concomitantly with doxycycline. Photosensitivity occurred more frequently in patients treated with doxycycline for 14 or 15 days (16/750 [2.1%], 95% CI 1.2–3.4%) than in those treated for 10 days (0/108 [0%], 95% CI 0.0–3.4%), but the difference was not significant (*P* = 0.24). Neither did this difference reach statistical significance when comparing 14-day (8/488 [1.6%], 95% CI 0.7–3.2%) versus 10-day group (*P* = 0.36) and 15-day (8/262 [1.3–5.9%], 95% CI 1.2–3.4%) versus 10-day group (*P* = 0.11).

**Table 2:** Number of patients with erythema migrans and frequency of photosensitivity reactions according to month of enrolment/presentation

Month	Jan	Feb	Mar	Apr	May	Jun	Jul	Aug	Sep	Oct	Nov	Dec	Overall
Number of patients	6	0	3	5	26	123	251	178	139	76	34	17	858
Patients (%) with photosensitivity	0	0	0	0	0	3 (2.4%)	10 (4.0%)	0	2 (1.4%)	1 (1.3%)	0	0	16 (1.9%)

## Discussion

In the present analysis, 16/858 (1.9%) patients with EM treated with doxycycline experienced photosensitivity reactions. The clinical symptoms of photosensitivity ranged from itching and burning sensations to mild erythema of sun-exposed face and extremities. A particular strength of our study is that we had data on a large number of patients with the same clinical entity who were treated at a single medical centre and given the same instructions, thus avoiding potential confounders when comparing data from different centres not using uniform approaches.

The frequency of doxycycline-induced photosensitivity found in our study is at the lower limit of some earlier reports [5, 7–10, 13]. The reason for much higher incidence rates (up to 15%) [4, 6, 11] reported in some other studies is not known. It was suggested that higher daily dose of doxycycline (300 mg) used for prolonged time (20 days) may have contributed to the higher frequency of these reactions [3, 4]. However, some other studies with lower daily dosages and shorter treatment duration found similarly high incidence rates of doxycycline-induced photosensitivity [5, 11, 12].

Another possible explanation for the differences encountered could be variations in exposure to the sun. This is supported by the highest frequency of photosensitivity reactions during the July, which is the month with the highest average monthly hours of sunshine in Slovenia. Accordingly, we found that 13/16 patients who developed photosensitivity did not follow the recommendation to avoid sun exposure. However, since we obtained data on sun exposure only in patients who reported photosensitivity but not in those who did not experience this adverse event, we could not evaluate the magnitude of the impact of sun exposure for development of photosensitivity. This is a major limitation of our study. The second limitation of our study is the fact that exact timing of the onset of photosensitivity reactions for the majority of patients experiencing photosensitivity was not obtained.

Interestingly, we observed photosensitivity more frequently in patients treated with doxycycline 100 mg twice daily for 15 days than in those treated for 14 days, and not at all in the 10-day treatment group. However, overlapping 95% CIs suggest that these differences were not significant. This accords with earlier findings that doxycycline-induced photosensitivity might depend on the dose of doxycycline and the intensity of UV-A radiation [17], but is not related to duration of therapy [18].

We found that women were more often affected than men (13/483 [2.7%] versus 3/387 [0.8%]; *P* = 0.04). It is not clear why in our study the women were affected more often than men. After carefully reviewing previous studies no differences in overall proportion of females were found, however exact data on sex proportion among patients with photosensitivity reactions in these studies were not provided (Table 1). Sex and/or other inherent/genetic characteristics may predispose an individual to photosensitivity. However, data to support this assumption are scarce [19]. Another possibility may be that in our study the women were more frequently exposed to the sun.

The previous studies on doxycycline efficacy in patients with EM did not provide detailed information on clinical symptoms of photosensitivity [3–14]. In our cohort of patients the clinical symptoms of photosensitivity were mild and had no long-term sequelae which may not always be the case when using doxycycline [15].

When deciding on which of the recommended antibiotics to prescribe for treating a patient with EM, several aspects should be considered: efficacy; drug allergy; adverse effects, including phototoxicity; pharmacokinetic/pharmacodynamic properties; ecological effect on the microbiota; likelihood of co-infection with *A. phagocytophilum*, which, if suspected would favour the use of doxycycline; and cost. Doxycycline has better central nervous system penetration than other oral antibiotics recommended for treatment of EM and remains the only oral antibiotic with proven favourable treatment outcome in patients with early Lyme neuroborreliosis [2]. Doxycycline may have other advantages over β-lactam antibiotics, such as reduced potential to cause *Clostridium difficile* infection [20], and is associated with low probability of allergic reactions [21]. In addition, since the antimicrobial spectrum of doxycycline is not limited to Lyme borreliae, observations of this study may support the use of doxycycline in other tick-borne diseases, including rickettsioses [22].

## Conclusions

When deciding on antibiotic treatment in adult patients with EM, the potential phototoxicity of doxycycline need not be regarded as a major drawback. This study showed that if patients were advised to restrict exposure to the sun, such adverse events occurred only exceptionally and exhibited no long-term sequelae.

## Abbreviations

CI: Confidence intervals
EM: Erythema migrans
IQR: Interquartile range
